# 

**Published:** 1933

**Authors:** 


### CONTENTS.

| | Page |
|---|---|
| Surgery for Pain. By Ernest Rock Carling, F.R.C.S. | 81 |
| Thirty Years' Progress in the Study of Rheumatic Heart Disease. By the late Carey F. Coombs, M.D., F.R.C.P. | 93 |
| Familial Multiple Telangiectases of the Skin and Mucous Membranes. By Gordon R. Scarff, M.B., Ch.B., F.R.C.S. | 113 |
| The Need for a Standard Method of Estimating the Blood-Pressure. By G. Arbour Stephens, M.D. | 121 |
| Reviews of Books | 127 |
| Editorial Notes | 134 |
| Obituary. David Samuel Davies, M.D., LL.D. | 135 |
| Meetings of Societies | 138 |
| The Medical Library of the University of Bristol | 142 |
| Publications Received | 145 |
| Local Medical Notes | 147 |

